# Case report: Capillary hemangioma in the renal hilum mimicking paraganglioma

**DOI:** 10.3389/fonc.2022.1027157

**Published:** 2022-11-08

**Authors:** Weixing Jiang, Xiaoqi Liu, Li Wen

**Affiliations:** ^1^ Department of Urology, Beijing Friendship Hospital, Capital Medical University, Beijing, China; ^2^ Department of Urology, National Cancer Center, National Clinical Research Center for Cancer, Cancer Hospital, Chinese Academy of Medical Sciences and Peking Union Medical College, Beijing, China

**Keywords:** capillary hemangioma, renal hilum, paraganglioma, case report, mass

## Abstract

**Background:**

Capillary hemangioma is a common benign tumor in children. Its presence in the kidney is rare, and there have been only case reports in the English literature. Herein, we report a special case of capillary hemangioma located in the renal hilum, which was suspected to be a paraganglioma.

**Case presentation:**

A 44-year-old woman had an irregular mass in the right hilar region. She had a history of hypertension for 3 years, and her 24-hour urinary norepinephrine was slightly high (41.53 µg, normal range: 16.69-40.65 µg). Abdominal MRI revealed a mass in the renal hilum measuring approximately 4.8×4.0×3.2 cm, slightly low signal intensity on T1WI, and very high signal intensity on both T2WI and DWI. The multiphase enhanced scan showed that the tumor had obvious enhancement with a central hypointense area. Therefore, paraganglioma was initially diagnosed. Phenoxybenzamine was administered over the next 2 weeks. She performed laparoscopic right hilar area tumor resection, and the kidney was preserved. Unexpectedly, the final pathology report was capillary hemangioma.

**Conclusions:**

Capillary hemangioma in the renal hilum is extremely rare. Surgery is the first choice to reduce the risk of compression symptoms and to rule out malignancy with respect to an undefined growing retroperitoneal mass. In addition, renal-sparing surgery should be preferred.

## Introduction

Capillary hemangioma is the most common benign tumor in infancy and early childhood and is also known as infantile hemangioma. It is a congenital tumor or vascular malformation commonly found in the skin and soft tissues and is caused by the proliferation of vasculogenic cells during the embryonic period (1, 2). Its presence in the kidney is rare, and there have been only case reports in the English literature (3, 4). Renal hemangiomas are usually solitary and unilateral. Preoperative diagnosis of renal hemangioma is very difficult because there are no specific radiologic characteristics (5). Thus, it is difficult to distinguish from kidney cancer, and this may result in unnecessary nephrectomy procedures. Capillary hemangioma is a type of hemangioma that consists of a network of dilated and hyperplastic capillaries. To our knowledge, capillary hemangioma that occurs in the renal hilum is even rarer, and 3 cases have been reported in the English literature (6–8).

In this report, we describe our experience with a 44-year-old woman who underwent surgery due to a suspected paraganglioma, but a histopathological study of the specimen showed capillary hemangioma.

## Case presentation

**Figure 1:**
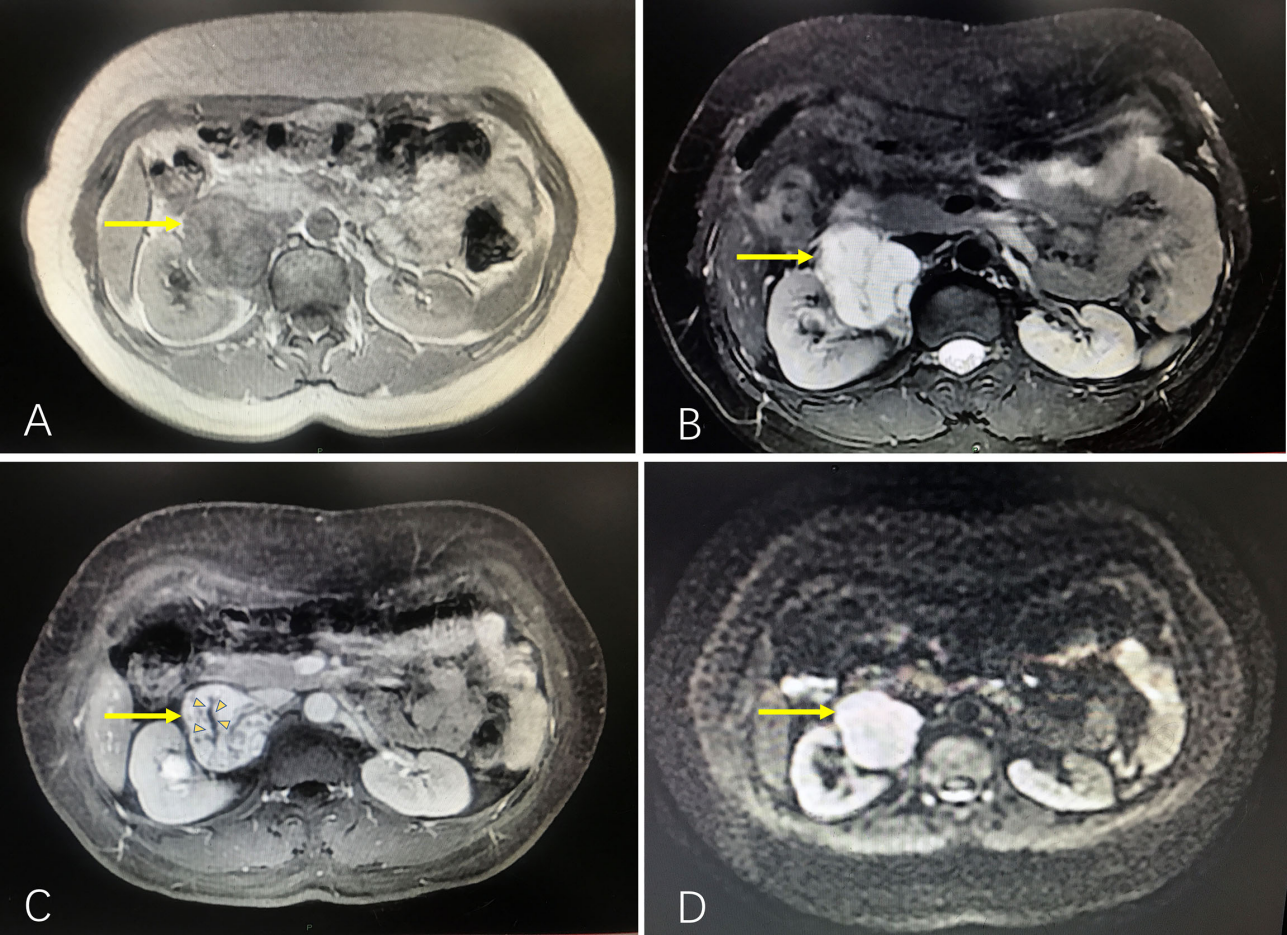
Abdominal MRI showing the mass compressing the right renal pelvis and ureter, and the right renal arteriovenous and inferior vena cava were also slightly compressed. **(A)** T1WI, low signal similar to the abdominal aorta (arrow). **(B)** T2WI, high signal for tumor (arrow). **(C)** Contrast-enhanced scan revealing that the tumor had an obvious enhancement with a central hypointense area (small arrow heads), and the filling of the contrast agent from the edge to the center was delayed. **(D)** DWI, high signal for the tumor (arrow).

A 44-year-old woman was admitted to our hospital with a mass that was found by physical examination and was present in the right renal hilar region. Ultrasonography showed a solid mass in the renal hilum measuring 4.2×3.1 cm. She had no complaints of paroxysmal headache, dizziness, heart palpitations, lumbago, gross hematuria, or other discomfort, but she had a history of hypertension for 3 years. The physical examination at admission showed that her weight was 50 kg, her heart rate was 80 beats per minute, her blood pressure was 143/95 mmHg (1 mmHg=0.133 kPa), and there was no tenderness or percussion pain in either of her kidneys. The preoperative laboratory tests showed a 24-hour urinary free cortisol level of 25.06 µg (normal value range, 12.3-103.5 µg), a 24-hour urinary norepinephrine level of 41.53 µg (normal range, 16.69-40.65 µg), a 24-hour urinary adrenaline level of 2.5 µg (normal range, 1.74-6.42 µg), and a 24-hour urinary dopamine level of 168.9 µg (normal range, 120.93-330.59 µg). Other hormones, including adrenocorticotropic hormones, renin, potassium, aldosterone, and sex hormone levels, were normal. Additionally, a blood test showed a hemoglobin concentration of 142 g/L and a hematocrit level of 40% (normal 35%-45%). She underwent abdominal MRI, which revealed an irregular mass in the right hilar region measuring approximately 4.8×4.0×3.2 cm, slightly low signal intensity on T1WI similar to the abdominal aorta, and very high signal intensity on both T2WI and DWI. The multiphase enhanced scan showed that the tumor had obvious enhancement with a central hypointense area, and the filling of the contrast agent from the edge to the center was delayed. The mass compressed the right renal pelvis and ureter, and the right renal arteriovenous and inferior vena cava were also slightly compressed (**Figure 1**). In combination with the above manifestations, the possibility of paraganglioma (ectopic pheochromocytoma) was considered. Thus, phenoxybenzamine (10 mg, twice a day) was administered.

**Figure 2:**
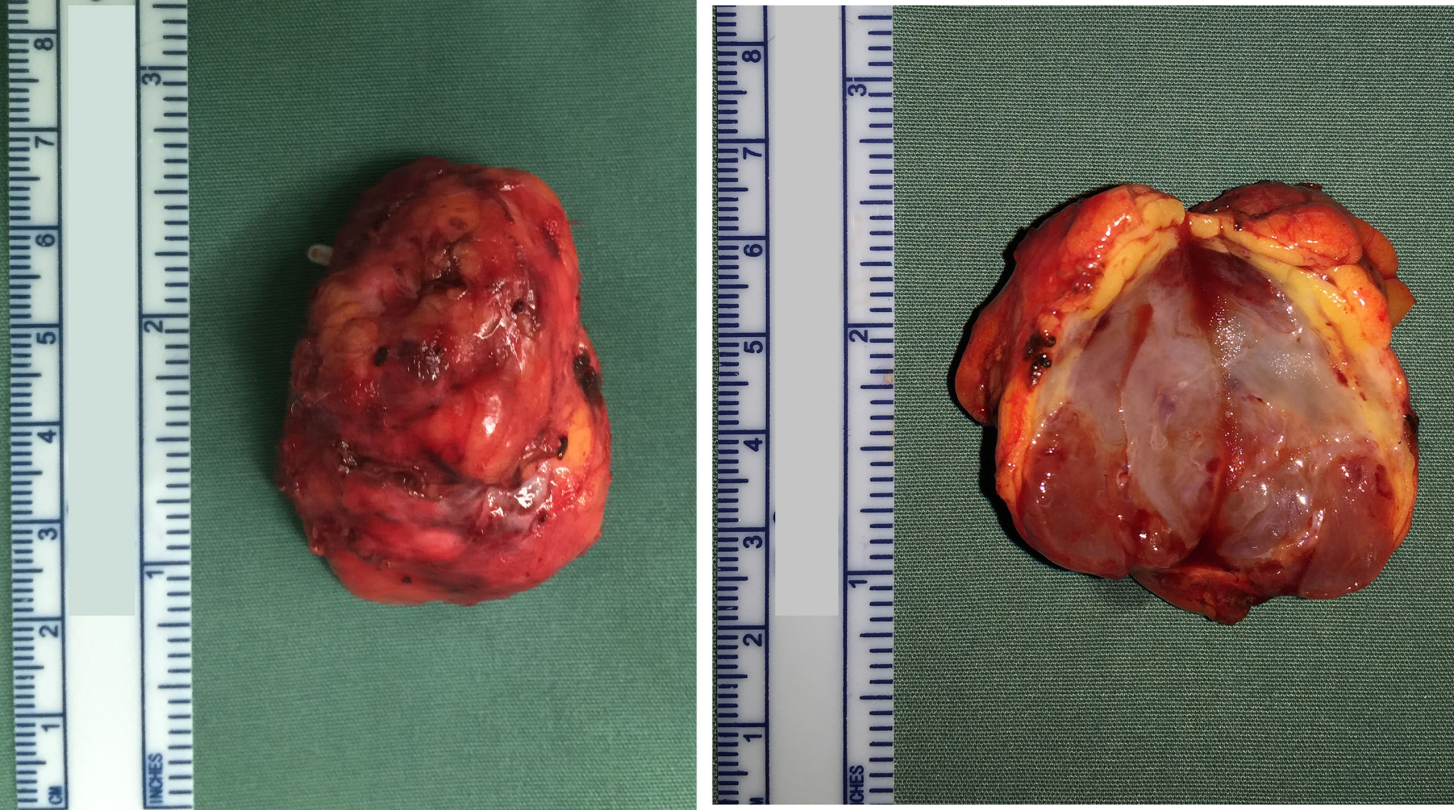
The gross specimen shows a well-defined solid mass of a white and brown color, and the cut surface was slightly jelly like.

**Figure 3:**
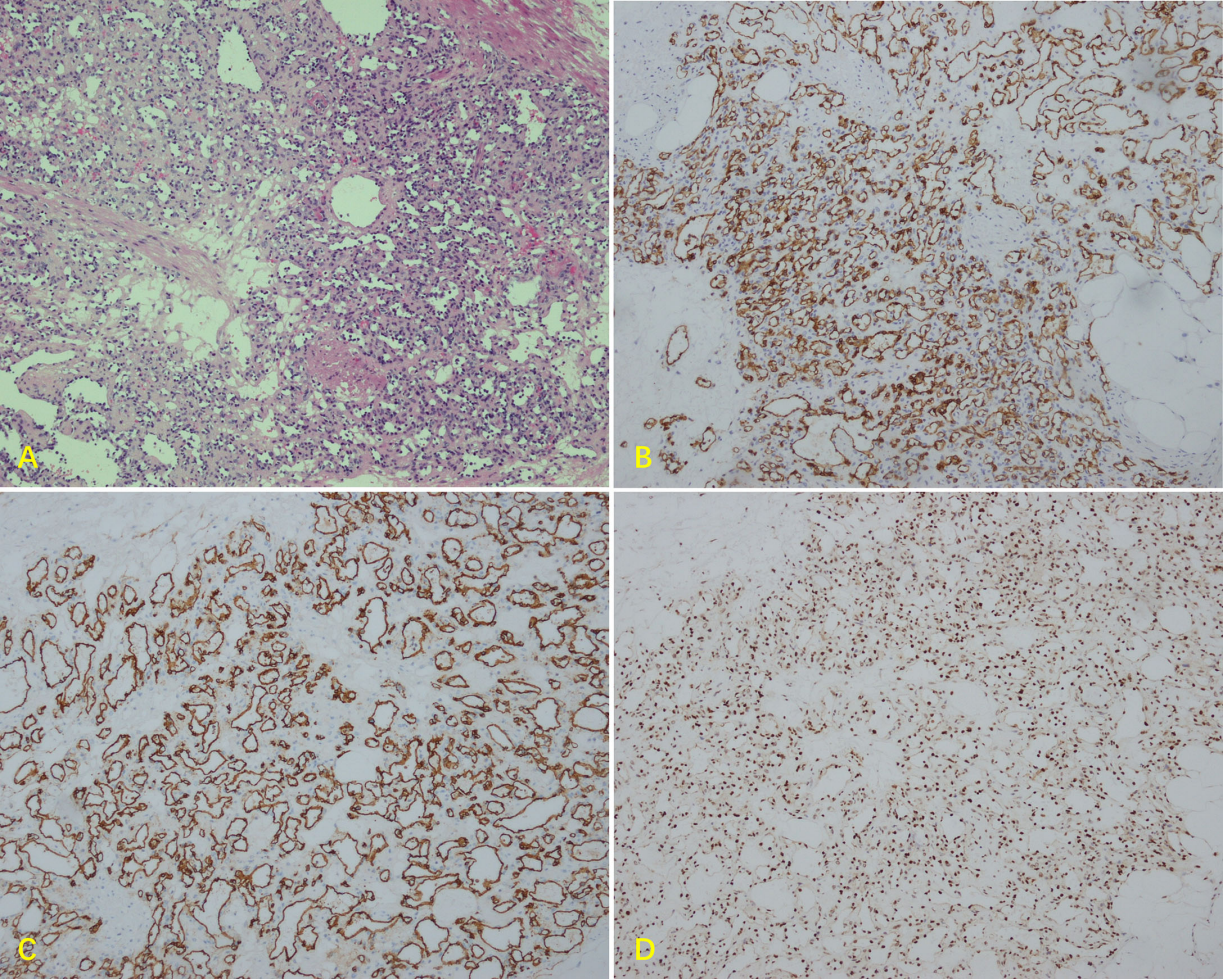
Microscopic findings of the renal hilum tumor. **(A)** Paraffin section showing the mass with lobulated capillaries (original magnification x100). Immunohistochemistry showing diffuse strong positivity for **(B)** CD31, **(C)** CD34, and **(D)** Fli1.

After 2 weeks, the patient had a blood pressure of 100/68 mmHg, a body weight of 52 kg and hematocrit was 33.1%; she also complained of mild nasal congestion. Therefore, she underwent transperitoneal laparoscopic surgery in a 60° lateral position. The kidney was preserved, and the surgery was successfully performed in 2 hours. During the surgery, the tumor was carefully separated from the kidney, renal hilar vessels, and inferior vena cava. The patient’s blood pressure remained stable throughout surgery. The estimated blood loss was approximately 400 ml. The source of blood loss was primarily the blood vessels around the tumor. Several hem-o-locks were used to control bleeding. We also tried to block the renal artery temporarily (within 15 minutes), which was partly effective. The specimen received in the pathology department showed that the mass was nodular, and its greatest diameter was measured to be approximately 4 cm. The mass was mostly solid and was white and brown in color. The cut surface was slightly jelly like with a medium texture, and the boundary was still clear (**Figure 2**). Microscopically, there were lobulated capillaries covered by a monolayer of endothelial cells, red blood cells in the lumen, no smooth muscle fibers in the perivascular wall and no fibrous connective tissue between the vessels (**Figure 3A**). No significant cytologic atypia was identified. The immunohistochemical results were as follows: CD31 (3+), CD34 (3+), Fli1 (3+), Ki-67<5% (+), AE1/AE3 (-), ChrA (-), CD56 (-), Syno (-), NSE (-), S-100 (-), HMB-45 (-), Melanoma Pan (-), Melan-A (-), and EMA (-). Positive immunohistochemical results are shown in **Figure 3**. The final pathology report was capillary hemangioma. The recovery period was uneventful, and she was discharged from the hospital 7 days after the operation. Abdominal CT was performed to rule out recurrence 3 months and 1 year after surgery. The patient had no recurrence of the tumor. However, hypertension (between 145/90 mmHg and 150/95 mmHg) was observed in a recent follow-up. We have referred her to the hospital for blood pressure control.

## Discussion

Capillary hemangiomas are most commonly found in children and can occur throughout the body. Hemangiomas that occur in the oral and maxillofacial area account for 60% of systemic hemangiomas, and the next most common locations are the trunk and extremities (9). Most of these tumors occur in the skin, subcutaneous tissue, and oral mucosa, such as the tongue, lips, bottom of the mouth and other tissues. They may also occur in internal viscera, such as the liver, spleen, gastrointestinal tract (10), omentum (11), and kidney (4).

Capillary hemangiomas are benign tumors that commonly do not affect normal life. However, the specific site of hemangioma is prone to serious complications, such as an intracranial hemangioma rupture causing intracerebral hemorrhage and an intestinal hemangioma rupture causing gastrointestinal hemorrhage. According to the published literature, there have been less than 40 cases of renal capillary hemangioma reported (12). There have also been reports of renal rupture and bleeding (4). Therefore, these conditions must be treated in a timely manner. In several studies, researchers have reported cavernous hemangiomas located at the renal hilum (13–15), but capillary hemangiomas are seldom reported. One capillary hemangioma case was close to the renal artery, and this patient was preoperatively misdiagnosed as having a pseudoaneurysm (6). One patient had tight adhesion to the renal vein, and this patient underwent radical nephrectomy (7). The third case is a lobular capillary hemangioma with synchronous ipsilateral renal cell carcinoma (16). Surgery is the most effective treatment for ectopic capillary hemangiomas, and active surveillance may also be an optional treatment scheme for special cases, such as solitary kidney. As we know, a pathologic diagnosis of capillary hemangioma after surgical excision is not challenging, but an accurate preoperative diagnosis is difficult. CT and MRI are commonly used methods, but in some locations, it is difficult to distinguish capillary hemangiomas from common masses or malignancies.

The patient in this study had a special case—capillary hemangioma at the hilar site of the kidney was different from the other reported cases. First, this patient had a larger tumor diameter. Second, this patient had a history of hypertension before surgery, mildly abnormal findings on her endocrine tests, and high enhancement on enhanced MRI. Considering the safety of the operation, we first prepared the patient by administering medications for 2 weeks. Then, we performed tumor resection and preserved the right kidney. The mass was accidentally diagnosed as a capillary hemangioma after surgery. Postoperatively, all imaging was reviewed to assess whether a preoperative diagnosis could have been made. Several subdivided bifurcated vessels could barely be observed during the enhanced scan phase of MRI. However, paraganglioma or malignancies are also hypervascular. This evidence was insufficient to draw conclusions. Enhanced CT may show the presence of little enhancement which has been sustained into the delayed phase (5). Regretfully, CECT was not performed in this patient preoperatively. If capillary hemangioma is considered, CECT could be a preferred diagnostic method. Based on the first impression of ultrasonography, our primary diagnosis was renal cell carcinoma. To further define the nature of the mass, MRI is the first choice to obtain a definitive diagnosis and to differentiate benign diseases from malignant diseases (including histological types). The MRI results showed that the mass was not from the kidney, and a PGL was considered. Finally, our focus was ectopic pheochromocytoma.

The growth rate of renal capillary hemangioma is still not clear because only cases have been reported. However, we insist that they must not be ignored in this case. First, surgical excision is often performed for all cases of renal capillary hemangioma, as the imaging appearance is similar to that of malignant lesions. Second, possible rupture of hemangioma could be dangerous. Finally, surgery could reduce the risk of compression symptoms in the future, especially in this case.

## Conclusion

Capillary hemangioma in the renal hilum is extremely rare and is difficult to accurately diagnose preoperatively. However, excision is appropriate in highly vascular tumors to reduce the risk of compression symptoms in the future and to rule out malignancy with respect to an undefined growing retroperitoneal mass. In addition, treatment options that include renal-sparing surgery should be preferred.

## Data availability statement

The original contributions presented in the study are included in the article/supplementary material. Further inquiries can be directed to the corresponding author.

## Ethics statement

The studies involving human participants were reviewed and approved by Cancer Hospital Chinese Academy of Medical Sciences (ID:20/245-2441). The patients/participants provided their written informed consent to participate in this study.

## Author contributions

All authors listed in this manuscript contributed significantly to the study. WJ contributed to writing the manuscript. XL contributed to the pathological diagnosis. LW contributed to reviewing the manuscript for critical revisions. All authors contributed to the article and approved the submitted version.

## Conflict of interest

The authors declare that the research was conducted in the absence of any commercial or financial relationships that could be construed as a potential conflict of interest.

## Publisher’s note

All claims expressed in this article are solely those of the authors and do not necessarily represent those of their affiliated organizations, or those of the publisher, the editors and the reviewers. Any product that may be evaluated in this article, or claim that may be made by its manufacturer, is not guaranteed or endorsed by the publisher.
